# A case report of arsenic-induced peripheral neuropathy misdiagnosed as Guillain-Barré syndrome

**DOI:** 10.3389/ftox.2026.1722489

**Published:** 2026-02-05

**Authors:** Shanshan Fu, Guanao Wu, Yibo Zhan, Hui Xia, Chang Zhou, Jingjing Zeng, Ye Tang, Boyan Pan, Zequan Zheng, Min Zhao, Yuanqi Zhao, Haoyou Xu

**Affiliations:** 1 The Second Clinical College, Guangzhou University of Chinese Medicine, Guangzhou, Guangdong, China; 2 Department of Neurology, The Second Affiliated Hospital of Guangzhou University of Chinese Medicine, Guangdong Provincial Hospital of Chinese Medicine, Guangzhou, China

**Keywords:** arsenic poisoning, Guillain-Barré syndrome, Mee’s lines, peripheral neuropathy, traditional medicine and alternative medicine

## Abstract

**Objective:**

To explore the clinical characteristics and misdiagnosis causes of arsenic-induced peripheral neuropathy.

**Methods:**

We report a case of arsenic-induced peripheral neuropathy initially misdiagnosed as Guillain-Barré syndrome, with analysis of clinical characteristics, diagnostic workup and therapeutic management, supplemented by literature review.

**Results:**

A 58-year-old male presented with progressive peripheral neuropathy, initially diagnosed with Guillain-Barré syndrome but later suspected as chronic inflammatory demyelinating polyneuropathy. However, his symptoms progressed despite standard immunotherapy, developing generalized motor deficits, myokymia, ascending sensory loss, and facial palsy. Careful reevaluation revealed critical diagnostic clues: a history of intermittent topical application of an unidentified herbal medicine preceding symptom onset, accompanying gastrointestinal prodromal symptoms, and characteristic physical findings including Mee’s lines, lower limb hyperpigmentation, and foot hyperkeratosis. Subsequent laboratory testing confirmed markedly elevated arsenic levels in nail and hair samples, ultimately establishing the diagnosis of arsenic-induced peripheral neuropathy.

**Conclusion:**

Arsenic-induced peripheral neuropathy can clinically mimic Guillain-Barré syndrome. For immunotherapy-refractory cases, clinicians should maintain high suspicion for potential heavy metal poisoning. Careful elicitation of toxic exposure history and recognition of characteristic dermatologic findings are critical for definitive diagnosis, as early recognition significantly improves prognosis.

## 1 Introduction

Arsenic, an amphoteric metal with versatile compound applications, poses risks of both acute and chronic poisoning when improperly used. With improved state regulation of hazardous chemicals, occupational arsenic poisoning and endemic arsenic poisoning are becoming increasingly rare, and food-borne arsenic poisoning is even rarer. However, medicinal use such as topical applications in rural areas may cause iatrogenic poisoning. Peripheral neuropathy is one of the cardinal clinical manifestations of arsenic poisoning, yet current understanding of this condition remains limited both domestically and internationally. Our hospital admitted a patient who was initially misdiagnosed with Guillain-Barré syndrome (GBS) and then suspected to have chronic inflammatory demyelinating polyradiculoneuropathy (CIDP), until finally confirmed as arsenic-induced peripheral neuropathy (AIPN) after over 2 months. This article will systematically summarize the clinical characteristics of AIPN and its differentiation from GBS based on the present case.

## 2 Case report

**FIGURE 1:**
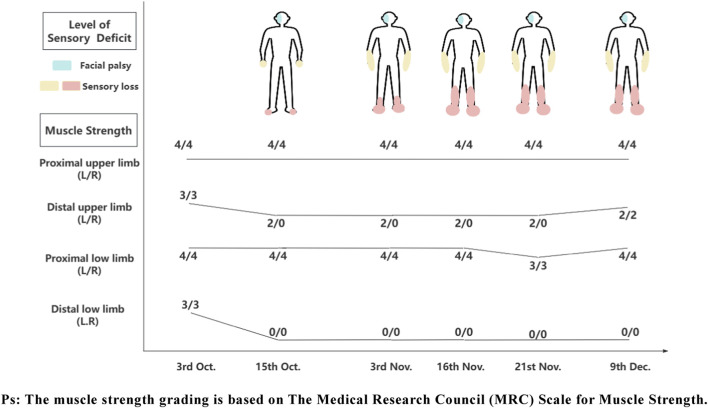
The patient’s physical signs.

A 58-year-old male began to experience weakness and decreased sensation in both lower limbs from 30 September 2022, manifested as easily losing slippers and difficulty in walking, so he visited Hospital A on October 3. Upon admission, physical examination revealed proximal limb strength of grade 4 and distal limb strength of grade 3 (Figure 1). MRI of the brain and spinal cord showed no abnormal signal intensities. Lumbar puncture results indicated normal intracranial pressure, slight elevation of total protein and IgG in cerebrospinal fluid (CSF), absence of oligoclonal bands in both serum and CSF, and no bacterial growth in CSF. Testing for 24 peripheral neuropathy antibodies in serum showed weak positivity for anti-sulfatide antibody (Table 1). Then he was diagnosed with GBS and treated with intravenous immunoglobulin (IVIG) and methylprednisolone. During treatment, the patient developed new-onset right-sided facial palsy, along with neuropathic pain and sensory loss distal to the wrists and ankles in all four limbs in addition to his initial symptoms. Consequently, he was referred to a tertiary hospital for further treatment.

**TABLE 1:** Diagnostic and therapeutic timeline since disease onset.

Time	Laboratory examination and imaging examination	Electrophysiological examination	Treatment
Hospital A (3rd -15th Oct.)	• Laboratory tests Intracranial pressure: 140 cmH_2_O (80–180cmH_2_O) Total CSF protein: **548.00 mg/L** (150–450 mg/L) CSF IgG: **73.70 mg/L** (10.0–30.0 mg/L) CSF WBC count:0/µL CSF cryptococcus neoformans bacteria + bacterial smear: no bacteria found 24 peripheral neuropathy antibodies in serum^a^: Weak positive for anti-sulfatide antibody (BLOT method) • Imaging studies Cranial + spinal cord MRI: no obvious lesions or demyelinating disease found	Not available	1. IVIG^d^ (10 g, ivd, qd, 5 days) 2. Methylprednisolone (480 mg, ivd, qd, 3 days)
Hospital B (3rd -16th Nov.)	• Laboratory tests CSF paraneoplastic antibodies^b^: Negative Ranvier antibodies^c^: Negative Pan’s test for CSF: **Positive (2+)** CSF protein:**2064.1 mg/L** (120–600 mg/L) CSFalbumin:**1070.00 mg/L** (0.00–350.00 mg/L) CSF WBC count:0/µL CSF Immunoglobulin A:**92.5 mg/L** (1–6 mg/L) CSF Immunoglobulin M:**5.64 mg/L** (0.00–1.00 mg/L) CSF Immunoglobulin G:**383.00 mg/L** (10–60 mg/L) Immunoprotein fixed electrophoresis, cerebrospinal fluid bacterial culture, and tumor markers: Normal	• Nerve conduction studies 1. CMAPs and SNAPs were unrecordable in most tested nerves, except for a low-amplitude right ulnar motor response and a normal left musculocutaneous motor study 2. F-waves were absent • Needle EMG Fibrillation potentials and positive sharp waves at rest were observed in the right abductor digiti minimi, right vastus medialis, and left tibialis anterior muscles, indicative of denervating injury • Blink reflex testing Prolonged latencies or absence of the R1 and R2 waves bilaterally • Blink reflex testing Prolonged latencies of left R1 and R2 with absent left R2’; absent right R1 and R2 with prolonged latency of right R2’	1. IVIG (25 g, ivd, qd, 5 days) 2. Methylprednisolone (1000 mg, ivd, qd, 3 days) 3. Neurotrophic medications (unknown)
Our hoapital (21st Nov. - 9th Dec.)	• Laboratory tests 24 peripheral neuropathy antibodies in serum^a^: Negative Procollagen III N-terminal peptide: **19.6 ng/L** (0–15.0 ng/L) Laminin:**151.2 ng/L** (0–130.0 ng/L) Type IV collagen:**132.6 ng/L** (0–95.0 ng/L) Hyaluronic acid:**312.1 ng/L** (0–120.0 ng/L) Urine mercury <0.10 ug/L Urine arsenic <0.17 umol/L Urine lead <1.3 ug/L Arsenic content in fingernails: **3.12 mg/kg** Arsenic content in hair: **7.60 mg/kg**	• Nerve conduction studies 1. No recordable CMAPs or SNAPs were obtained in all tested limbs 2. F-waves were unelicitable in bilateral median and ulnar nerves 3. No H-reflexes detected in bilateral tibial nerves • Needle EMG Fibrillation potentials and fasciculation potentials were observed in the bilateral tibialis anterior muscles, bilateral first dorsal interossei, right deltoid, and extensor digitorum communis muscles, indicative of denervating injury • Blink reflex testing Universally prolonged wave latencies with asynchronous R1 responses • Repetitive nerve stimulation No significant increment or decrement in amplitude was observed in the orbicularis oculi muscles	1. Sodium thiosulfate (1.28 g, ivd, qd, 16 days) 2. Methylprednisolone (36 mg, ivd, qd. 10 days +32 mg, ivd, qd, 3 days) 3. Mouse nerve growth factor (18ug, ivd, qd, 15 days) 4. Vitamin B1 (10 mg, po, tid, 19 days) and mecobalamin (0.5 mg, im, qd, 19 days) 5. Pregabalin capsule (75 mg, po, bid, 6 days) 6. Compound glycyrrhizin tablets (50 mg, po, tid, 16 days)

^a^ Anti-sulfatide antibody, anti-GM1, antibody, anti-GM2 antibody, anti-GM3, antibody; anti-GM4 antibody, anti-GD1a antibody, anti-GD1b antibody, anti-GD2, antibody; anti-GD3 antibody, anti-GT1a antibody, anti-GT1b antibody, anti-GQ1b antibody.

^b^ Anti-Hu antibody IgG, anti-Ri antibody IgG, anti-CV2, antibody IgG, anti-ANNA-3, antibody IgG, anti-PCA-2, antibody IgG.

^c^ Anti-NF155, IgG4, anti-NF186, IgG.

^d^ The patient’s weight was 50 kg.

Abnormal results are shown in bold.

After discharge, the patient experienced exacerbation of limb weakness and numbness, accompanied by limb muscle atrophy. He subsequently visited Hospital B on November 3. Upon admission, physical examination revealed decreased distal limb strength compared to before, elevated sensory loss levels, and absent tendon reflexes in all four limbs. Admission tests showed negative results for paraneoplastic and anti-Ranvier antibodies in CSF. Routine CSF analysis was positive for the Pandy test, with significant elevation of total protein, IgA, IgM, and IgG. Immunofixation electrophoresis and tumor markers were all within normal limits. Electrophysiological examination indicated the presence of a severe, asymmetric polyradiculoneuropathy. Its predominant feature is an axonal pattern of injury with associated demyelinating features, involving motor and sensory nerves in all four limbs as well as cranial nerves. Considering the possibility of CIDP, the doctors administered neurotrophic medications, methylprednisolone, and intravenous IVIG for treatment. At the time of his release on November 16th, physical examination showed further progression of sensory loss levels in both lower limbs.

**FIGURE 2:**
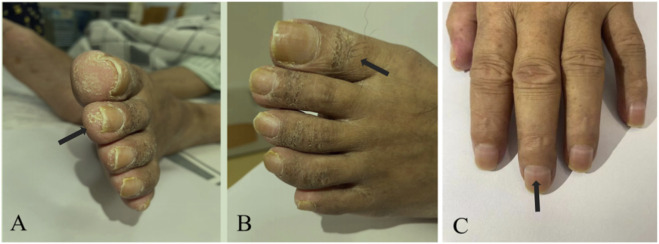
The patient’s typical cutaneous changes. Hyperkeratosis on the toes. **(A)** Hyperpigmentation. **(B)** Mees’ lines on the nails **(C)**.

On November 21, the patient was admitted in our hospital via wheelchair. Neurological examination revealed grade 3 proximal muscle weakness in both lower limbs. Cutaneous findings included bilateral toe and heel hyperkeratosis, pigmentation below the knees, and Mees’ lines on the nails (Figure 2). Liver fibrosis panel like laminin were elevated, while liver function tests are unremarkable. Repeat testing of the serum peripheral neuropathy antibody panel was negative, in contrast to the initial positive finding. Electrophysiological studies revealed a predominantly axonal pattern of injury in the limbs, as evidenced by needle EMG findings. Additionally, the blink reflex studies were consistent with a demyelinating process. Toxicology screening demonstrated elevated arsenic concentrations in nail and hair levels (Chakraborti et al., 2003). The ongoing clinical progression and refractoriness to standard immunotherapy led us to consider alternative diagnoses other than typical immune-mediated disorders like GBS or CIDP. Consequently, based on the characteristic dermatological manifestations and confirmed arsenic levels, we established arsenic poisoning as the definitive cause of the peripheral neuropathy. During exposure source tracing, the patient reported intermittent use of topical hemorrhoid medication from a local clinic prior to symptom onset and presented with persistent nausea, vomiting, and anorexia for 1 week. Then the attending physician contacted the clinic to verify the hemorrhoid treatment’s composition, but arsenic testing was unfeasible due to logistical constraints after the clinic denied using arsenic-containing drugs.

The patient was treated with sodium thiosulfate for detoxification, mouse nerve growth factor for nerve nutrition, methylprednisolone for anti-inflammatory therapy, and other symptomatic therapy. Discharged on 9 December 2022, the patient exhibited restored muscle strength (grade 2 in bilateral upper limb distal regions and grade 4 in bilateral lower limb proximal regions), with other conditions unchanged. Through active rehabilitation, the patient achieved grade 3 muscle strength in the bilateral upper limb distal and lower limb proximal regions by December 28, while distal lower limb strength showed no significant improvement.

## 3 Discussion

Arsenic oxides and salts are highly toxic and can be absorbed via the gastrointestinal tract, respiratory tract, and skin. Hepatic methylation reduces toxicity before elimination. While oral intake is the main route in non-occupational poisoning, dermal absorption is often overlooked. Thus, misuse of arsenic-containing topical medications may cause iatrogenic poisoning. Our patient developed peripheral neuropathy, initially misdiagnosed as GBS, and was presumed to be related to topical arsenic exposure.

### 3.1 Arsenic-induced peripheral neuropathy

The toxic effects of arsenic are primarily mediated through its high affinity for sulfhydryl groups (Ratnaike, 2003). This binding not only disrupts cellular oxidative phosphorylation, leading to reduced ATP synthesis, but also forms unstable ADP-arsenate complexes that further compromise energy metabolism (Mathew et al., 2010). Given that enzymes containing sulfhydryl groups are widely distributed throughout various systems in the human body, arsenic compounds can cause damage to multiple organ systems.

AIPN is a neurological disorder caused by exposure to arsenic and its compounds, typically manifesting 1–3 weeks after poisoning. It is characterized by symmetrical distal sensorimotor dysfunction. Beyond peripheral limb nerves, cranial nerves including the olfactory, optic, glossopharyngeal, vagus, and vestibulocochlear nerves may also be affected (Yan et al., 2002; Zeyu et al., 1998). But the pathophysiological differences between cranial and peripheral nerve damage require further investigation.

The prognosis of AIPN is critically dependent on the dose and duration of toxic exposure, as well as the timeliness of therapeutic intervention. A cross-sectional study in Bangladesh linked chronic arsenic-contaminated water to dose-dependent sensory neuropathy, with irreversible neuronal injury typically occurring after 5–10 years (Lu et al., 2017). While early detoxification reduces arsenic levels and improves outcomes, delayed treatment especially exceeding 6 months often results in only partial symptom relief, with 20%–33% of patients developing permanent motor or sensory deficits due to axonal degeneration (Chhuttani et al., 1967; Lagerkvist and Zetterlund, 1994).

### 3.2 Distinction between AIPN and GBS

#### 3.2.1 Disease course patterns

The two conditions exhibit distinct temporal profiles. GBS can present with an acute or subacute onset, usually reaching peak severity within 4 weeks, and demonstrates self-limiting progression with favorable prognosis in most cases (Shahrizaila et al., 2021). The clinical course and prognosis of arsenic poisoning depend on the duration and dose of toxic exposure.

#### 3.2.2 Clinical manifestations

While both disorders may present with symmetrical limb weakness, sensory disturbances and facial paralysis, their prodromal features offer critical diagnostic clues. In early arsenic poisoning, patients may exhibit gastrointestinal manifestations such as nausea, vomiting, oral burning sensation, and watery or bloody diarrhea (Huang and Liu, 2010; Su et al., 2002). While some GBS patients may experience prodromal gastrointestinal symptoms, these are predominantly diarrhea, particularly in cases associated with *Campylobacter* jejuni infection (Elendu et al., 2024).

#### 3.2.3 Laboratory findings

The cerebrospinal fluid protein-cell dissociation phenomenon can support the diagnosis of GBS, but the its specificity is not high, as this phenomenon is also seen in patients with AIPN (Donofrio et al., 1987). Serum anti-ganglioside antibodies serve as key diagnostic indicators for GBS, detectable in 30%–60% of patients with prevalence closely correlating to clinical subtypes. However, these antibodies are more frequently detected in variant forms of GBS, while their prevalence is relatively lower in the classic form (Willison et al., 2016).

#### 3.2.4 Neurophysiological examination

Typical GBS are primarily characterized by demyelinating changes, while certain variants predominantly feature axonal damage. In contrast, AIPN predominantly involves axonal injury, though some studies suggest that segmental demyelination may be the primary pathological feature in its early stages (Donofrio et al., 1987; Kim et al., 2012). Consequently, it can be easily misdiagnosed as GBS (Berbel-Garcia et al., 2004; Donofrio et al., 1987; Goddard et al., 1992; Perriol et al., 2006; Wu et al., 2013). It is noteworthy that in a subset of AIPN cases, motor or sensory nerve action potentials may be unrecordable (Xing et al., 2007). This electrophysiological characteristic is potentially influenced by the patient’s baseline health status, as well as the duration and dosage of arsenic exposure. Future cases should include detailed records for comparative analysis. In such cases, spontaneous potentials on needle EMG, such as fibrillations, provide key diagnostic evidence, which was pivotal in our case.

#### 3.2.5 Characteristic findings

AIPN typically presents with a clear history of arsenic exposure and systemic manifestations, such as abnormal liver function tests and cutaneous signs like hyperkeratosis or Mees’ lines (Sharma et al., 2016). However, these clues are often overlooked in practice. A key diagnostic feature, as exemplified in this case, is its lack of response to immunotherapy. When arsenic poisoning is suspected, the diagnosis can be confirmed by demonstrating elevated arsenic levels in any biological tissue. It is important to note that the clinical interpretation varies by sample type: serum arsenic reflects recent exposure and declines rapidly, 24 h urinary arsenic is ideal for diagnosing acute poisoning and monitoring chelation, while hair and nail measurements are more indicative of chronic exposure (Ratnaike, 2003).

### 3.3 Heavy metal poisoning caused by improper treatment with traditional medicine and alternative medicine

A 1977-2023 review of traditional and complementary medicine (T&CM)-related heavy metal poisoning revealed Asia, North America, and Europe as primary regions, with the US, India, and China constituting beyond 50% of cases. Furthermore, significant safety concerns were noted: 48.5% of contaminated products had untraceable origins, while the remainder involved uncertified T&CM practitioners. This distribution was concentrated in medically underserved regions (Gerke and Seifert, 2025). Additionally, 3.4% of poisoning cases resulted from dermal exposure, and instances of poisoning via rectal administration were also documented (Gerke and Seifert, 2025; Mitchell-Heggs et al., 1990; Wu et al., 2013). Therefore, even when the exact source of exposure remains unidentified, arsenic levels in human tissues remain a crucial diagnostic criterion for arsenic poisoning when considered alongside specific clinical manifestations, as demonstrated in the presented case.

## 4 Conclusion

Arsenic poisoning-induced peripheral neuropathy is often misdiagnosed as GBS. Clinicians should comprehensively collect patients’ prodromal symptoms and medication history when assessing peripheral neuropathy. For patients who sought traditional medicine treatments prior to onset, it is essential to investigate the components of the medications used. Characteristic skin manifestations and multisystem involvement can serve as important diagnostic clues for arsenic poisoning. Patients with suspected AIPN should undergo arsenic testing in blood, urine, hair, and nails, if available, with the selection of specimens guided by the rate of symptom progression.

## Data Availability

The original contributions presented in the study are included in the article, further inquiries can be directed to the corresponding authors.
